# Determination of the Loss of Function Complement *C4* Exon 29 CT Insertion Using a Novel Paralog-Specific Assay in Healthy UK and Spanish Populations

**DOI:** 10.1371/journal.pone.0022128

**Published:** 2011-08-03

**Authors:** Lora Boteva, IMAGEN, Yee Ling Wu, Josefina Cortes-Hernández, Javier Martin, Timothy J. Vyse, Michelle M. A. Fernando

**Affiliations:** 1 Division of Molecular Medicine and Genetics and Division of Immunology, Infection and Inflammatory Disease, Guy's Hospital, King's College London, London, United Kingdom; 2 Center for Molecular and Human Genetics, The Research Institute at Nationwide Children's Hospital, Columbus, Ohio, United States of America; 3 Autoimmune Disease Research Unit, Vall d'Hebron University Hospital Research Institute, Universitat Autonoma, Barcelona, Spain; 4 Instituto de Parasitologia y Biomedicina “Lopez-Neyra”, CSIC, Granada, Spain; University of Hawaii Manoa, United States of America

## Abstract

Genetic variants resulting in non-expression of complement *C4A* and *C4B* genes are common in healthy European populations and have shown association with a number of diseases, most notably the autoimmune disease, systemic lupus erythematosus. The most frequent cause of a *C4* “null” allele, following that of *C4* gene copy number variation (CNV), is a non-sense mutation arising from a 2 bp CT insertion into codon 1232 of exon 29. Previous attempts to accurately genotype this polymorphism have not been amenable to high-throughput typing, and have been confounded by failure to account for CNV at this locus, as well as by inability to distinguish between paralogs. We have developed a novel, high-throughput, paralog-specific assay to detect the presence and copy number of this polymorphism. We have genotyped healthy cohorts from the United Kingdom (UK) and Spain. Overall, 30/719 (4.17%) individuals from the UK cohort and 8/449 (1.78%) individuals from the Spanish cohort harboured the CT insertion in a *C4A* gene. A single Spanish individual possessed a *C4B* CT insertion. There is weak correlation between the *C4* CT insertion and flanking MHC polymorphism. Therefore it is important to note that, as with *C4* gene CNV, disease-association due to this variant will be missed by current SNP-based genome-wide association strategies.

## Introduction

The human complement *C4* locus, located in the class III region of the major histocompatibility complex (MHC) on the short arm of chromosome 6, is highly polymorphic and exhibits genetic complexity. Complement *C4* genes show segmental duplication as part of mono-, bi-, tri-, or quadri-modular RCCX cassettes. RCCX modules comprise the following four genes encoded in tandem - ***R*** *P1* ( now known as *STK19*), cytochrome P450 steroid-21 hydroxylase (***C*** *YP21A2*), complement ***C*** *4*, and tenascin B (*TN* ***X*** *B*). Two to eight copies of *C4* genes may be present in a diploid human genome; with each chromosome 6 comprising 1 to 4 copies of a *C4* gene. The *C4* gene exists as either of two paralogs: *C4A* (acidic) (MIM: 120810) or *C4B* (basic) (MIM: 120820), each of which is polymorphic in itself. At the nucleotide level, *C4A* and *C4B* share 99% sequence homology over 41 exons. Each paralog is defined by five nucleotide changes in exon 26, which contribute to four isotype-specific amino acid residues from positions 1120 to 1125: **PC**PV**LD** for C4A and **LS**PV**IH** for C4B [1], [2]. In addition, *C4* genes may vary in size depending on the presence (21 kb) or absence (14.6 kb) of an intron 9 HERV-K(C4) insertion resulting in long and short forms, respectively.

Genetic variants resulting in non-expression of *C4A* and *C4B* genes (so-called “null” alleles, C4A*Q0 and C4B*Q0) are common in healthy European populations and have shown association with a number of diseases, most notably the autoimmune disease, systemic lupus erythematosus (SLE/lupus; MIM: 152700) [3], [4], [5], [6]. The majority of *C4A* or *C4B* “null” alleles arise as a consequence of copy number variation (CNV) at the *C4* locus where either paralog is absent from a particular haplotype. For example, some MHC haplotypes harbour mono-modular RCCX cassettes which comprise either *C4A* or *C4B*. Hence, the alternate *C4* paralog will be physically absent and not expressed at transcript or protein level. Other *C4* null alleles result from non-sense mutations in *C4A* and *C4B* genes leading to the absence of the respective protein. The most frequent cause of a *C4* null allele, following that of CNV, is a non-sense mutation arising from a 2 bp CT insertion into codon 1232 of exon 29 in *C4*. This causes a frameshift change in the *C4* gene which alters the subsequent amino acid sequence and results in a termination codon in exon 30 [7]. Additional non-sense mutations that have been described in the literature tend to show restriction to rare families demonstrating complete homozygous C4 deficiency: these include a G to A substitution at the donor site of the intron 28 splice junction, a 1 bp deletion in exon 20, a 2 bp deletion in exon 13, a 1 bp deletion in exon 13, a C to T transition in exon 13, and a 4 bp insertion in exon 36, on a variety of haplotypic backgrounds [7], [8], [9].

In the past, the presence of *C4* null alleles was inferred from the absence or reduced level of C4A or C4B proteins from serum or plasma. Partial deficiency of C4A or C4B described the phenomenon by which one isotype was expressed at about half the level of the other. The absence or unequal serum/plasma protein levels of C4A and C4B can be caused by the physical absence or non-sense mutations of the corresponding gene, the unequal number of *C4A* and *C4B* genes in a diploid genome, and differential protein expression levels governed by the presence of long or short *C4* genes. Therefore, such “immunophenotyping” methods are not accurate measures of genetic variation at the *C4* locus.

As previously mentioned the non-sense mutation resulting from a CT insertion into exon 29 of the *C4* gene is a frequent cause of C4 deficiency. Previous attempts to accurately genotype this polymorphism have not been amenable to high-throughput typing, have been confounded by failure to account for CNV at this locus, as well as inability to distinguish between *C4A* and *C4B* [7], [10], [11], [12], [13], [14], [15]. We have developed a novel, high-throughput, paralog-specific assay to determine the presence and copy number of the exon 29 CT insertion within the *C4* gene using nanogram quantities of DNA. For the first time, we have examined the frequency, SNP-CNV correlation and HLA association of this polymorphism in healthy cohorts from the United Kingdom and Spain. We have integrated these data with that of total complement *C4* gene copy number (GCN), *C4A* GCN and *C4B* GCN using our previously published paralog ratio test (PRT) [16].

## Methods

### Study Cohorts

#### 1958 British Birth Cohort

719 unrelated subjects from the 1958 British Birth Cohort that had been previously genotyped to high-density at the MHC using a custom Illumina panel were typed for the complement *C4* exon 29 *CT* insertion using our novel assay [17]. Two-digit *HLA-DRB1* genotypes were available for 661/719 (92%) of the subjects. The SNP and *HLA-DRB1* genotype data were used for SNP-CT insertion CNV correlation analyses.

#### UK Family Cohort

The inheritance of the complement *C4* exon 29 *CT* insertion was traced in 15 UK families, comprising 15 UK SLE probands who carried the insertion and 46 of their first degree relatives. All probands fulfilled the American College of Rheumatology criteria for SLE [18].

#### Spanish Control Cohort

The Spanish cohort comprised 460 healthy, unrelated individuals, including 253 subjects from Granada and 207 subjects from Barcelona. 2553 ancestry informative markers (AIMs) and 4179 SNP genotypes across the MHC region were available for all subjects. The samples from Granada were typed using a custom Illumina panel as part of the IMAGEN consortium study (manuscript in submission). The samples from Barcelona were typed using the Illumina 1 million OMNI-Quad chip. Four-digit *HLA-DRB1* data was available for 232/460 (50%) of the subjects. These data were used for SNP-CT insertion CNV analyses.

Informed written consent was obtained from all study participants.

This study was approved by the London Research Ethics Committee (Ref: 06/MRE02/9), United Kingdom, the Comité de Ética del CSIC, Granada, Spain and the Clinical Research Ethics Committee of Vall d'Hebron University Hospital, Barcelona, Spain.

### Assessment of population substructure and relatedness in Spanish cohort

All quality control analyses were performed separately for each cohort using PLINK except principal components analysis which was performed using EIGENSTRAT [19], [20]. Samples and SNPs were put forward for analysis if they met the following quality control filters: SNPs greater than 95% genotyping efficiency, MAF greater than 1%, samples greater than 95% genotyping efficiency and PI-Hat scores less than 0.2 on identity-by-state analysis using 2553 ancestry informative markers (AIMs) in order to exclude cryptic relatedness and duplicate samples. SNPs were excluded for deviation from Hardy-Weinberg equilibrium on the basis of a false discovery rate (FDR) of 0.05. In order to correct for population stratification, samples were excluded if they were outliers on principal components analysis using post-QC AIMs (performed using EIGENSTRAT and defined as greater than 6 standard deviations from the mean).

### Complement C4 paralog ratio test

All study individuals were typed for total complement *C4* gene copy number (GCN), *C4A* GCN and *C4B* GCN using our previously described paralog ratio test [16].

### Complement C4 exon 29 CT insertion Assay Design

**Figure 1:**
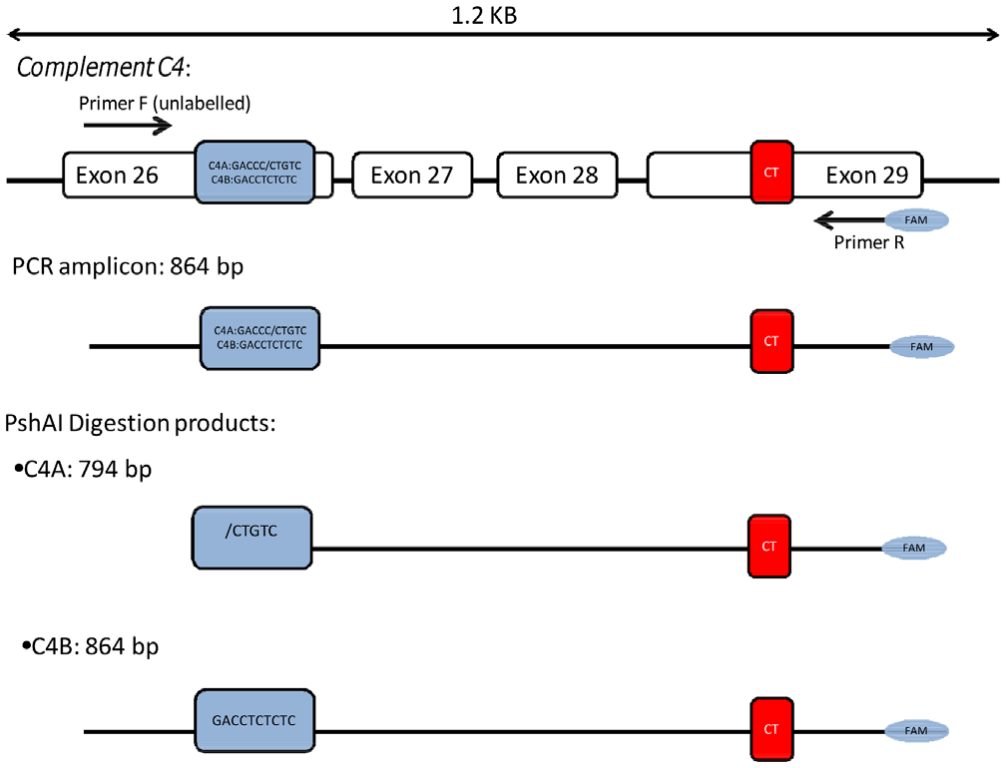
Schematic representation of paralog-specific complement *C4* exon 29 CT insertion assay design. The top panel illustrates exons 26 through to 29 of the complement *C4* gene. The paralog-specific nucleotide changes in exon 26 are shown in the blue box. The CT insertion in exon 29 is represented by the red box. The second panel depicts the FAM-labelled 864 bp amplicon obtained post-PCR. The third and fourth panels show the *C4A* and *C4B* paralog-specific digestion products respectively.

We based our assay on the principles of a restriction enzyme digest variant ratio analysis (REDVR). A PCR was designed to amplify a fragment spanning exon 26 of the *C4* gene, where the conserved, isotype-specific sequence differences are located, to exon 29, where the CT insertion is located (**Figure 1**). Following PCR amplification, the fragments were digested with *Psh*AI, a restriction enzyme recognising a site present within the paralog-specific sequence of *C4A* but not *C4B*. Digestion products were then separated using capillary electrophoresis, resulting in size-specific peaks for *C4A* (794 bp) and *C4B* (864 bp). The presence of the CT insertion in either of the two paralogs resulted in a peak shifted by two base pairs compared to the wild-type. For individuals heterozygous for the CT insertion in either *C4A* or *C4B*, a specific pattern of clearly separated double peaks was observed: the first peak corresponding to the normal copy(ies) and the second peak corresponding to the copy(ies) of the gene possessing the CT insertion (**Figure S1**). For individuals that were homozygous or hemizygous for the CT insertion, i.e. all copies of the *C4* gene harboured the CT insertion or all copies of a particular isotype, either *C4A* or *C4B*, harboured the CT insertion, a single peak shifted by two base pairs from the expected normal peak was seen. DNA from samples suggestive of homozygous or hemizygous states was mixed with DNA from the PGF cell line post PCR amplification. PGF is a MHC homozygous cell line with two normal copies each of *C4A* and *C4B*; therefore if the test sample was homozygous for the CT insertion in either/both *C4A* or *C4B*, the double peak pattern would be observed following capillary electrophoresis. The ratio of the area under the peak for insertion-positive and insertion-negative amplicons was used in combination with total *C4* gene copy number data to determine the number of *C4* copies carrying the insertion in an individual.

Specifically, for the initial PCR, the 864 bp fragment (spanning exon 26 to exon 29) was amplified using 25 ng (2.5 µl at 10 ng/µl) of genomic DNA and 2.5 µM forward primer, REDVRA_F (CTGAGAAACTGCAGGAGACATC) and 2.5 µM of 6-FAM labelled reverse primer, CTinsR ([6FAM]GACACGGCATTGCTCTGA). The reaction volume was 25 µl, comprising 2.5 µl of 10× PCR Buffer (QIAGEN), 0.5 µM of each dATP, dCTP ,dGTP and dTTP (Promega, Cat. No. U1240), 0.625 units of *Taq* DNA polymerase (Qiagen, Cat.No. 201205) and 14.375 µl of sterile water. PCR reaction conditions were: initial denaturation step at 94°C for 3 min, followed by 25 cycles at 94°C for 30 sec, 58°C for 30 sec and 72°C for 1 min, followed by a chase phase of 56°C for 1 min and 70°C for 20 min to ensure complete addition of the terminal adenine residue.

Following amplification, 10 µl PCR product was digested using 5 U of *Psh*AI restriction enzyme (NEB, Cat. No. R0593L) in 20 µl digestion reaction, comprising 2 µl 10×NEB Buffer 4, 0.2 µl 100× BSA and 7.3 µl of sterile water. The digestion reaction was incubated at 37°C for 1 hour. 3 µl of digested product mixed with 9.5 µl HiDi Formamide was denatured at 96°C for 3 minutes, placed on ice, and then analysed on a capillary sequencer (ABI 3730xl DNA analyzer, Applied Biosystems, using a 50 cm array) with 0.5 µl per sample of the marker LIZ1200 (Applied Biosystems Cat. No. 4379950). Following capillary electrophoresis, specific *C4A* and *C4B* peaks could be seen at 794 bp and 864 bp respectively (**Figure S1**).

All reactions were performed in 96-well plates. Each plate included six controls: four DNA samples from cell lines of known *C4* genotype (COX: total *C4* GCN = 2, *C4A* GCN = 0, *C4B* GCN = 0; QBL: total *C4* GCN = 2, *C4A* GCN = 2, *C4B* GCN = 0; PGF: total *C4* GCN = 4, *C4A* GCN = 2, *C4B* GCN = 2 and DBB: total *C4* GCN = 4, *C4A* GCN = 2, *C4B* GCN = 2), and two CEU samples from the same family: 1416-12 (NA 12249) and 1416-1 (NA 10835) which have been previously shown to harbour the CT insertion in a *C4A* gene [21] (ML Lokki, personal communication).

### Cloning

**Figure 2:**
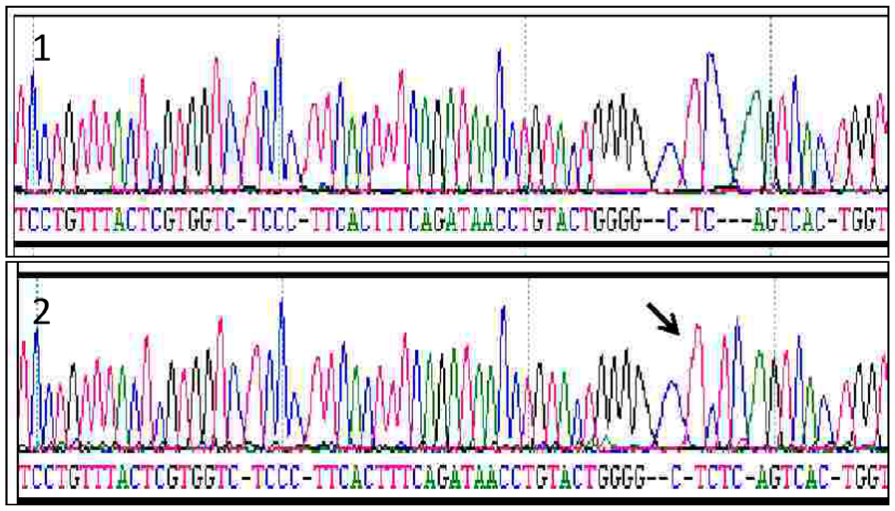
Cloned complement *C4* exon 29 sequence. Electropherogram demonstrating (1) normal complement *C4* exon 29 sequence and (2) complement *C4* exon 29 CT insertion (arrowed).

For validation, we cloned two samples harbouring CT insertions in *C4A* and *C4B* respectively, determined by our novel assay, using the TOPO TA Cloning® kit (Invitrogen, K4560-01). One sample had a homozygous CT insertion in *C4A* and the other possessed a heterozygous *C4B* CT insertion. For each sample, 4 µl of undigested PCR product was added to 1 µl of sterile water and 1 µl of TOPO® vector. The mix was incubated for 5 minutes at room temperature. 2 µl of the mix was added to a vial of electrocompetent One Shot TOP10 cells followed by electroporation (capacitance 25 µF, resistance 200 ohms, voltage 2.5 KV). 250 µl of SOC medium was added to the cells post-electroporation followed by an hour's incubation at 37°C and 200 rpm. The electroporated cells were spread on S-Gal LB agar plates containing 100 µg/ml ampicillin and incubated at 37°C overnight. Clear colonies were chosen and cultured overnight in 5 ml of LB Broth containing 50 µg/ml ampicillin. Plasmid DNA was extracted from the cells using the High Pure Plasmid Isolation Kit (Roche, Cat. No. 11 754 785 001). The cloned DNA was sequenced to confirm the presence of the CT insertion in the *C4* isotype of interest (**Figure 2**).

### SNP-C4 CT insertion CNV correlation

In order to determine the relationship between the complement *C4* exon 29 CT insertion and surrounding MHC polymorphism we calculated the correlation coefficient, r^2^, between SNP and *HLA-DRB1* genotypes and integer copy number estimates for the CT insertion in *C4A* using standard linear regression as described previously [16]. We used 2-digit *HLA-DRB1* data for 1958 British Birth Cohort subjects and 4-digit *HLA-DRB1* data for the Spanish control cohort. We used MHC region SNP data as previously described: 1230 SNPs for the UK cohort and 4179 for the Spanish dataset. We plotted the correlation coefficient, r^2^, against the chromosomal position of each SNP for *C4A* CT insertion copy number estimates. The size of the square representing each SNP is inversely proportional to the p-value. *C4B* CT insertion analyses were not performed as only a single Spanish individual possessed the polymorphism.

## Results

We have developed a novel, paralog-specific assay to detect the presence of a common complement C4 null allele: a 2 bp CT insertion in exon 29 of the complement *C4* gene. We have genotyped 719 United Kingdom (UK) individuals from the 1958 British Birth Cohort and 460 healthy Spanish individuals for the polymorphism using this assay. In addition, we determined total complement *C4* GCN, *C4A* GCN and *C4B* GCN in both cohorts using our previously described paralog ratio test [16].

### Healthy Spanish individuals display higher complement C4 gene copy numbers compared with healthy subjects from the United Kingdom

**Figure 3:**
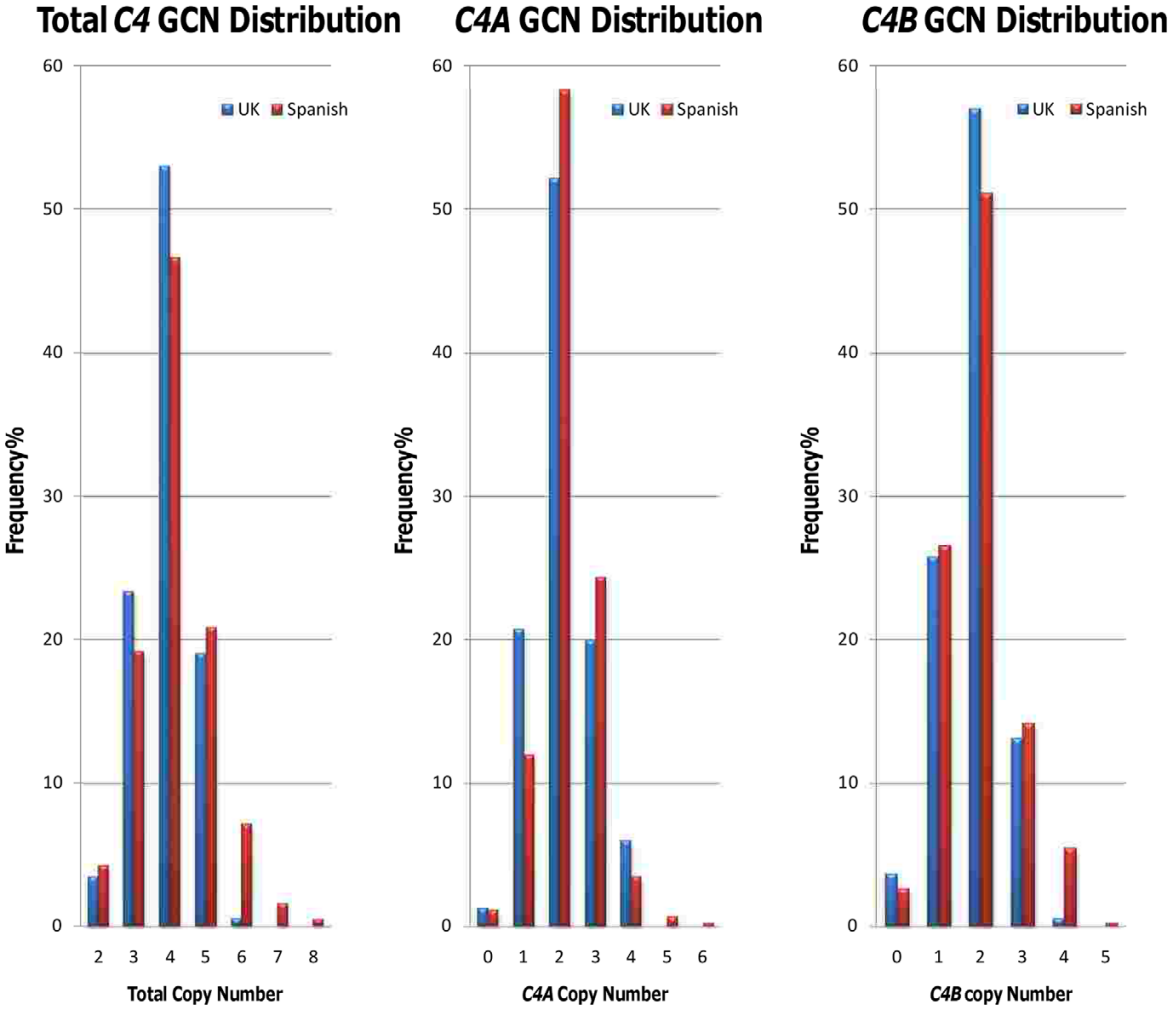
Complement *C4* gene copy number distributions in healthy United Kingdom and Spanish populations. From left to right, the histograms demonstrate total complement *C4* gene copy number (GCN), *C4A* GCN and *C4B* GCN distributions in healthy United Kingdom (blue bars) and Spanish (red bars) populations.

In the UK cohort, total *C4* GCN ranged from 2 to 6, with copy numbers from 0 to 4 observed for both *C4A* and *C4B* (**Figure 3**). Mean total *C4* GCN was 3.89 (±0.76), mean *C4A* GCN was 2.08 (±0.83) and mean *C4B* GCN was 1.81 (±0.72). The frequency of *C4A* null homozygotes was 1.25% while that of *C4B* null homozygotes was greater at 3.60%.

In the Spanish cohort, 11/460 individuals (2.39%) (9/207 individuals from Barcelona and 2/253 individuals from Granada) were removed due to relatedness or population outliers, resulting in a post-QC cohort size of 449 individuals. We observed a broader range of *C4* GCN estimates in this cohort compared with the UK cohort: total *C4* GCN varied from 2 to 8; *C4A* GCN from 0 to 6 and *C4B* GCN from 0 to 5 (**Figure 3**). Higher mean *C4* GCNs were observed in the Spanish dataset in comparison to the UK cohort: mean total *C4* GCN was 4.13 (SD±1.02) (p<0.0001), mean *C4A* GCN was 2.20 (SD±0.78) (p = 0.46) and mean *C4B* GCN was 1.94 (SD±0.86) (p = 0.11). The frequency of homozygous *C4A* null alleles (1.10%) was similar to that observed in the UK cohort (1.25%). However, homozygous *C4B* null alleles were observed less frequently in the Spanish (2.60%) compared with the UK cohort (3.60%).

### The complement C4 exon 29 CT insertion occurs more frequently in United Kingdom populations compared with Spanish populations

**Figure 4:**
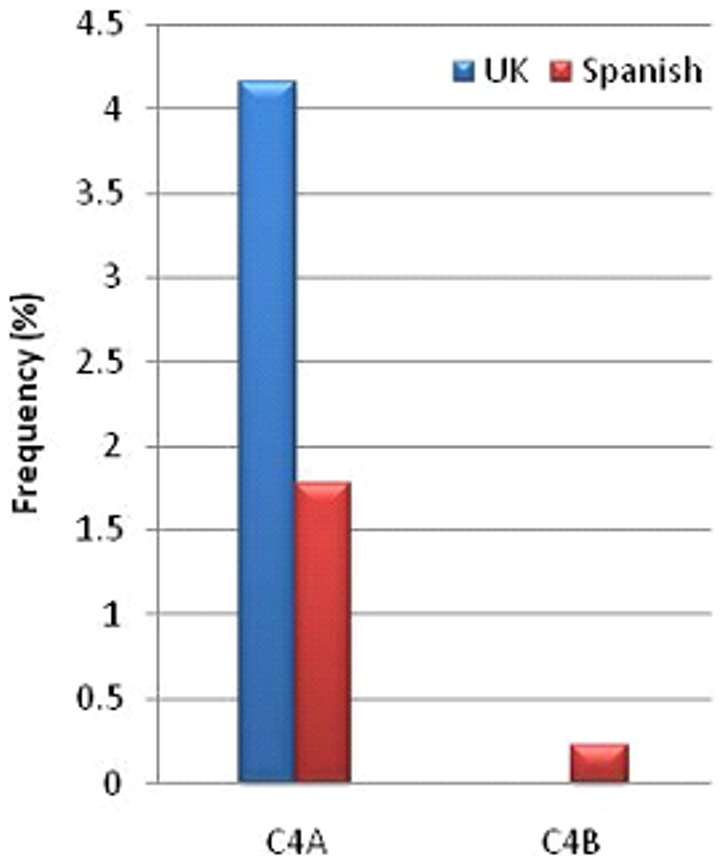
Frequency of the complement *C4* exon 29 CT insertion in healthy United Kingdom and Spanish populations. The histograms show the frequency of the complement *C4* exon 29 CT insertion in *C4A* and *C4B* in United Kingdom (blue bar) and Spanish (red bars) populations.

Overall, 30/719 individuals (4.17%) from the UK cohort harboured the CT insertion in *C4A* (**Figure 4**). 26 of 719 individuals (3.61%) harboured the CT insertion in a single *C4A* gene and at least one additional normal copy of the gene; 2/719 individuals (0.28%) possessed the CT insertion in two copies of *C4A* and one additional normal copy; a single individual (0.14%) had the insertion in three copies of *C4A* and a fourth, normal copy; a single individual (0.14%) had the insertion in the only copy of the gene, resulting in complete C4A deficiency. The frequency of chromosomes carrying a *C4A* CT insertion was 2.08%. CT insertions in *C4B* were not observed in the UK cohort.

Eight of the 449 individuals in the Spanish cohort (1.78%) possessed the CT insertion in a *C4A* gene, a much lower frequency than that observed in individuals from the UK (p = 0.02) (**Figure 4**). Six (1.34%) individuals had the insertion in one copy of *C4A* plus one normal copy of *C4A*. One (0.22%) individual had the insertion in one copy of *C4A* plus two normal copies of the gene, and one individual had the insertion in one copy of *C4A* plus three normal copies of *C4A*. We did not observe any individuals with complete C4A deficiency solely due to the presence of the exon 29 CT insertion. We did not detect any individuals with more than one copy of the CT insertion in *C4A* in the Spanish dataset, whereas in the UK cohort three individuals harboured more than one copy of the polymorphism, none of which occurred in conjunction with HLA-DRB1*13. It is unclear whether this observation is genuine or is consequent upon the larger size of the UK cohort. The frequency of chromosomes harbouring the *C4A* CT insertion was less than half that observed in the UK cohort at 0.89%. A single individual possessed a *C4B* CT insertion, in addition to a functional *C4B* copy, resulting in a frequency of chromosomes bearing *C4B* CT insertions of 0.11%.

### The complement C4 exon 29 CT insertion demonstrates Mendelian inheritance

**Figure 5:**
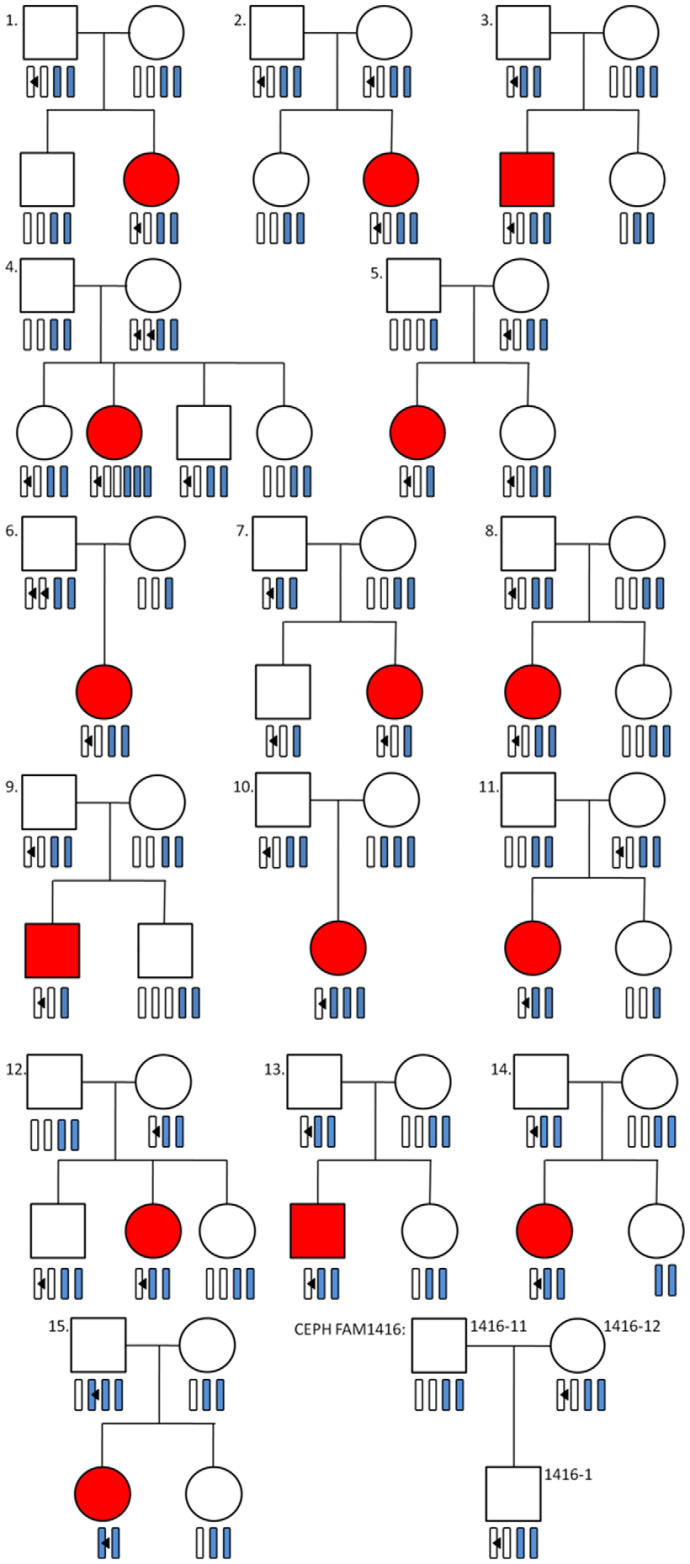
Mendelian inheritance pattern of the complement *C4* exon 29 CT insertion. The genetic pedigrees illustrate 15 SLE families where the SLE proband harbours a CT insertion together with a single CEU trio where mother and son are known to harbour the CT insertion in *C4A*. Open squares identify healthy males, open circles identify healthy females, red squares indicate SLE affected males and red circles indicate SLE affected females. Open bars indicate *C4A* genes and blue bars indicate *C4B* genes. Black triangles identify the presence of the *C4* exon 29 CT insertion.

In order to determine the inheritance pattern of the complement *C4* exon 29 CT insertion, we examined DNA from first degree relatives (parents and siblings) of 15 UK SLE probands known to harbour the *C4* CT insertion in either *C4A* or *C4B* (**Figure 5**). We did not find evidence of non-Mendelian inheritance in any of the families, further validating our method.

### The C4 exon 29 CT insertion shows strong linkage disequilibrium with HLA-DRB1*13

*HLA-DRB1* genotypes were available for 33 of the 39 samples carrying the CT insertion (84%): 28 of 30 UK samples (93%) and 5 out of 9 Spanish samples (55%). Overall, 78% of *C4* CT insertions were observed in individuals carrying at least one HLA-DRB1*13 allele. In the UK cohort, 82% (23/28) of individuals carried the CT insertion together with at least one DRB1*13 allele, while in the Spanish cohort 60% (3/5) of individuals carried the CT insertion in concert with at least one DRB1*13 allele, representing the greater haplotypic diversity of southern European populations or the smaller sample size of the Spanish cohort. No other *HLA-DRB1* alleles were over-represented among individuals carrying the polymorphism (**Table S1**).

### C4A CT insertion CNV-SNP correlation

**Figure 6:**
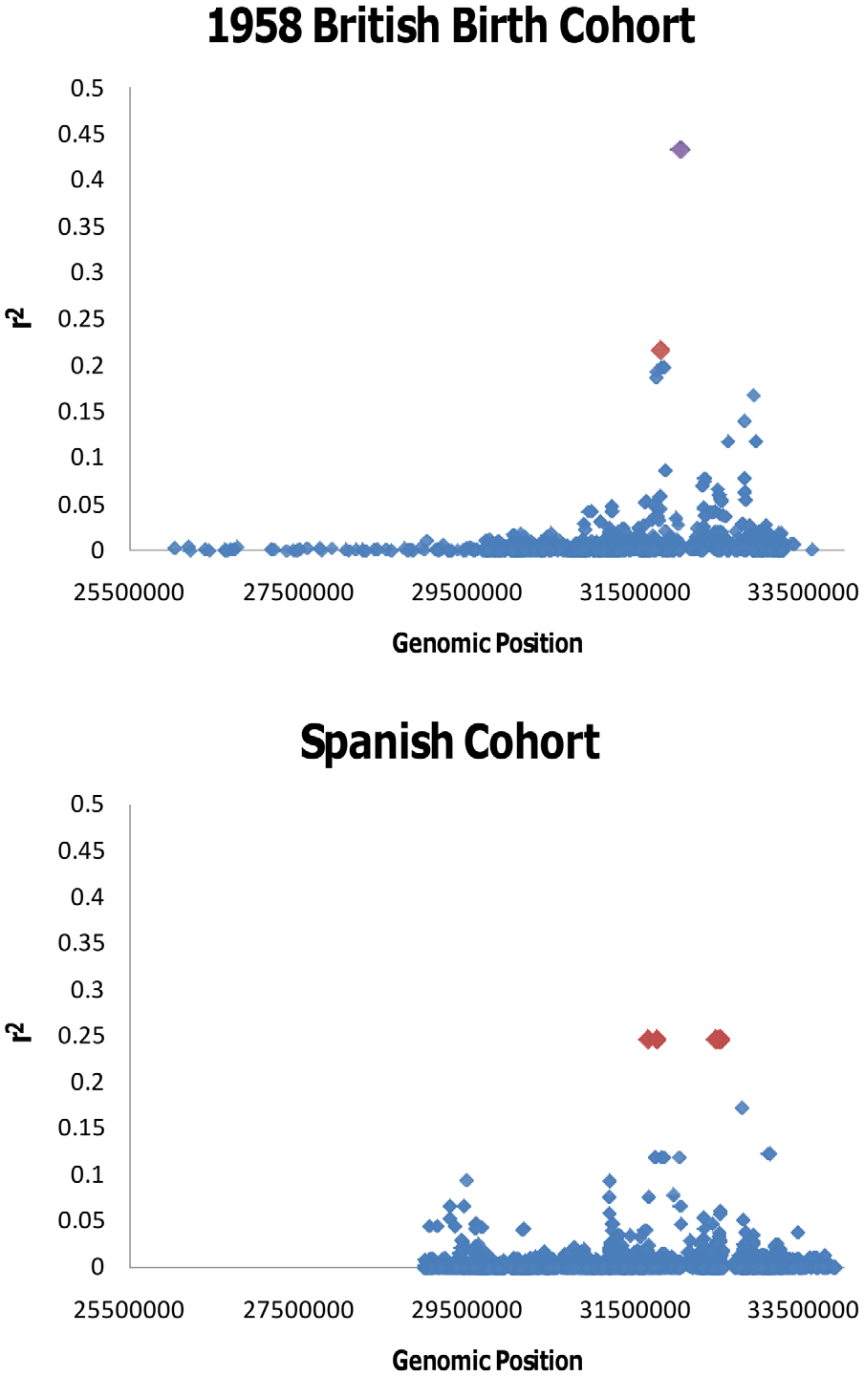
Correlation between *C4* exon 29 CT insertion and flanking MHC SNPs and *HLA-DRB1* alleles. The correlation coefficient, r^2^, between the *C4* exon 29 CT insertion and flanking MHC SNPs and *HLA-DRB1* alleles is shown. Data for the United Kingdom cohort is illustrated in the top panel while that for the Spanish cohort is shown in the bottom panel. The sizes of the diamonds are inversely proportional to the p values for each SNP or *HLA-DRB1* allele. Purple diamonds indicate r^2^>0.4, red diamonds indicate r^2^>0.2 but <0.4, blue diamonds indicate r^2^<0.2.

We used linear regression to assess correlation between the *C4A* CT insertion and surrounding SNPs and *HLA-DRB1* alleles in the UK and Spanish cohorts (**Figure 6** **and Table S2**). We did not find evidence of strong SNP-*C4A* CT insertion correlation.

In the UK dataset, the SNPs most highly correlated with the *C4A* CT insertion were located in the class III region of the MHC, telomeric to the RCCX module: *rs2734331* – a synonymous SNP in exon 11 of *SKIV2L* (r^2^ = 0.43), *rs453098* - a synonymous SNP in exon 2 of *C6orf25* (r^2^ = 0.20), *rs707915* in intron 5 of *MSH5* (r^2^ = 0.20) and *rs400547* in intron 5 of *CLIC1* (r^2^ = 0.20). The HLA-DRB1*13 allele has an r^2^ of 0.12 with the *C4A* CT insertion, demonstrating that while most *C4A* CT insertions appear on a HLA-DRB1*13 background, the majority of HLA-DRB1*13 haplotypes do not harbour the *C4A CT* polymorphism.

SNP-*C4A* CT insertion correlation values were lower in the Spanish compared with the UK cohort, reflecting the greater diversity of MHC haplotypes present in the Spanish population. The SNPs showing the greatest correlation were again seen in the class III region, telomeric and centromeric to the RCCX module: *rs2228088* - a synonymous SNP in exon 1 of *TNF* (r^2^ = 0.25) and *rs11964779* in intron 5 of *MSH5* (r^2^ = 0.12), *rs6927077* (r^2^ = 0.25), *rs6457580* (r^2^ = 0.25) and *rs6940690* (r^2^ = 0.25), all near *BTNL2*, as well as in the class II region of the MHC: *rs6902390* (r^2^ = 0.12) near *HLA-DPA1* and *rs10947368* - a synonymous SNP in exon 2 of *HLA-DOA* (r^2^ = 0.12).

The degree of correlation is such that neither SNPs nor *HLA-DRB1* alleles can be used as surrogate markers for the *C4A* exon 29 CT insertion.

## Discussion

We have developed a high-throughput, paralog-specific assay to detect the presence and copy number of an exon 29 complement *C4* CT insertion which results in non-expression of C4 at the protein level. This is important because C4 null alleles, inferred from immunophenotyping studies, have shown association with many disease states including the autoimmune disease, SLE. It is clear that complete homozygous deficiency of complement C4 is a strong genetic risk factor for SLE. However, due to the extended linkage disequilibrium shown by disease-associated haplotypes containing mono-modular RCCX cassettes in European populations, it remains to be determined whether partial C4 deficiency due to low functional *C4* gene copy number rather than complete deficiency of the protein is also an independent risk factor for SLE. If partial C4 deficiency is indeed important in disease susceptibility, then any genetic variant resulting in non-expression of C4 should also show association with disease. As the *C4* CT insertion occurs on haplotypic backgrounds that are different to those harbouring mono-modular RCCX cassettes, it should be possible to detect disease-association if the population frequency of the CT insertion is reasonable given an appropriately powered study.

Our UK frequency data for total *C4* GCN, *C4A* GCN and *C4B* GCN are consistent with previous European-American reports [22]. The Spanish data are novel and show higher total *C4* GCN compared to the UK cohort. As total complement *C4* GCN, *C4A* GCN and *C4B* GCN are greater in the Spanish cohort, the number of functional complement *C4* copies are also likely to be higher than northern European UK individuals.

The *C4* exon 29 CT insertion generally occurs on a HLA-DRB1*13 haplotypic background. The frequency of all HLA-DRB1*13 alleles is higher in the Spanish cohort (12.25%) compared to the UK dataset (10.44%). Interestingly, the frequency of the *C4* exon 29 CT insertion is lower in the Spanish cohort compared with the UK. This may be due to the greater variety of HLA-DRB1*13 haplotypes in the Spanish population.

We confirm that the *C4* CT insertion occurs almost exclusively in association with *C4A* on a HLA-A*02-HLA-C*03:04-HLA-B*40:01-HLA-DRB1*13(:02)-HLA-DQB1*06:04 haplotypic background, which was previously serologically defined as *HLA-B60-HLA-DR6* (**Table S1**) [7]. Following investigation of both cohorts, we found only a single Spanish individual with a CT insertion in *C4B*. These data are consistent with the CT insertion/mutation initially occurring on an ancestral *HLA-B60-C4A-HLA-DR6* haplotype. Subsequent recombination events have resulted in the presence of the polymorphism on diverse haplotypes. The rare occurrence of the mutation in *C4B* has been demonstrated in previous studies and most likely represents a gene conversion event [11], [12].

There is weak correlation between the *C4* CT insertion and flanking MHC polymorphism. Therefore it is important to note that, as with *C4* CNV, disease-association due to this variant will be missed by current SNP-based genome-wide association studies.

We plan to use this assay in conjunction with our PRT method to estimate functional complement *C4* gene copy number in SLE cohorts of differing ancestry in order to elucidate the association and independence of *C4* CNV in this genetically complex autoimmune disease.

## Supporting Information

Figure S1 **Capillary electrophoresis (Genescan) traces illustrating samples with and without the** ***C4*** **exon 29 CT insertion.**
(TIF)

Table S1 **HLA haplotypes for individuals harbouring complement** ***C4*** **exon 29 CT insertions.**
(PDF)

Table S2 ***C4A*** **CT insertion CNV-SNP correlation.**
(PDF)
