# Supplementary figures and images for: Determination of the Loss of Function Complement C4 Exon 29 CT Insertion Using a Novel Paralog-Specific Assay in Healthy UK and Spanish Populations
Source: PLoS One. 2011 Aug 3;6(8):e22128. doi: 10.1371/journal.pone.0022128 (PMC3153930; doi:10.1371/journal.pone.0022128)

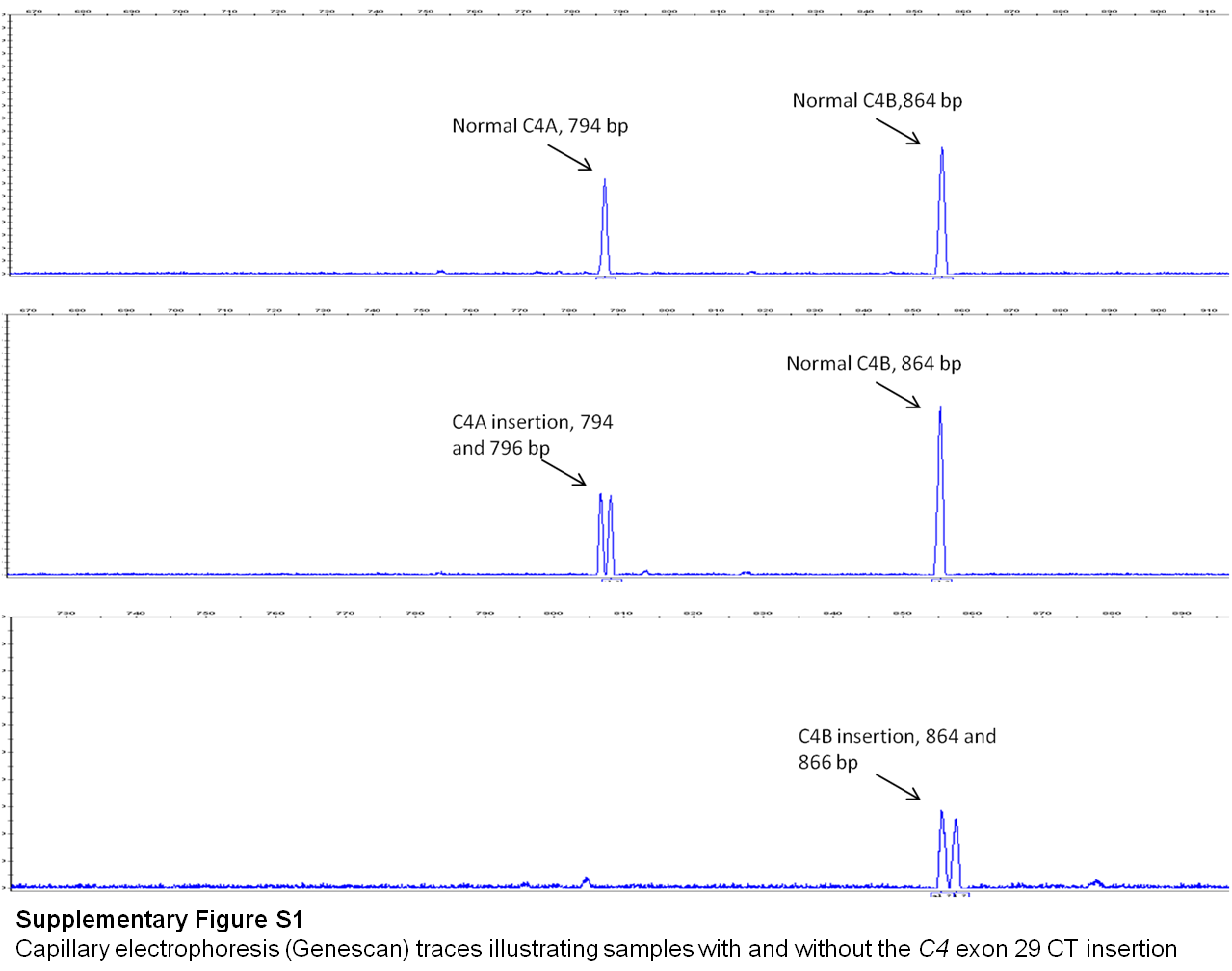

Supplement: Figure S1 — Capillary electrophoresis (Genescan) traces illustrating samples with and without the C4 exon 29 CT insertion. (TIF) [file pone.0022128.s001.tif]
