# Supplementary material for: Determination of the Loss of Function Complement C4 Exon 29 CT Insertion Using a Novel Paralog-Specific Assay in Healthy UK and Spanish Populations
Source: PLoS One. 2011 Aug 3;6(8):e22128. doi: 10.1371/journal.pone.0022128 (PMC3153930; doi:10.1371/journal.pone.0022128)
Supplement: Table S1 — HLA haplotypes for individuals harbouring complement C4 exon 29 CT insertions. (PDF) [file pone.0022128.s002.pdf]

Supplementary Table S1

HLA haplotypes for individuals harbouring complement *C4* exon 29 CT insertions

Sample	Cohort	Genotype†	Gene‡	*C4**	*C4A*[a]	*C4B*[b]	HLA-A_1	HLA-A_2	HLA-C_1	HLA-C_2	HLA-B_1	HLA-B_2	DRB1_1	DRB1_2	DQB1_1	DQB1_2
**BS2527**	Spanish	Het	C4A	3	2	1					B*4001	B*4403	*1302	*0701	*0604	*0202
**BS2538**	Spanish	Het	C4A	5	4	1					B*4201	B*4403	*0302	*0701	*0202	*0402
**BS2562**	Spanish	Het	C4A	4	2	2					B*4403	B*4901	*0404	*0701	*0202	*0302
**BS2717**	Spanish	Het	C4A	5	2	3							*1302	*0102	*0604	*0501
**BS2748**	Spanish	Het	C4B	4	2	2							*1305	*0101	*0301	*0501
**501_A07**	UK	Het	C4A	4	3	1	A*02	A*0101	C*0304	C*0702	B*4001	B*0702	*13	*0401	*0604	*0301
**501_B07**	UK	Het	C4A	5	2	3	A*02	A*6801	C*0501	C*0704	B*4402	B*4402	*13	*1201	*0604	*0301
**501_C03**	UK	Het	C4A	5	3	2	A*2301	A*2902	C*0602	C*0802	B*1402	B*3701	*13	*1001	*0604	*0501
**501_C06**	UK	Het	C4A	4	2	2	A*0101	A*2902	C*0304	C*1502	B*4001	B*5101	*13	*0404	*0604	*0302
**501_E04**	UK	Het	C4A	4	2	2	A*02	A*02	C*0304	C*0304	B*4001	B*1501	*13	*0401	*0604	*0302
**501_E06**	UK	Het	C4A	4	2	2	A*02	A*0301	C*0304	C*0401	B*4001	B*3501	*13	*0404	*0604	*0302
**501_H10**	UK	Het	C4A	5	3	2	A*02	A*3101	C*0304	C*0602	B*4001	B*1302	*13	*11	*0604	*0301
**502_A02**	UK	Het	C4A	4	2	2	A*2902	A*0301	C*0304	C*1601	B*4001	B*4403	*0701	*0701	*0202	*0303
**502_E10**	UK	Het	C4A	4	2	2	A*0101	A*6801			B*3701	B*4402	*13	*11	*0604	*0301
**601_D06**	UK	Het	C4A	4	2	2	A*02	A*2402	C*0304	C*0702	B*4001	B*3906	*13	*0801	*0604	*0402
**601_E02**	UK	Het	C4A	5	3	2	A*2501	A*2601	C*0304	C*0702	B*4001	B*0702	*1302	*1302	*0604	*0604
**601_E04**	UK	Het	C4A	4	2	2	A*02	A*3004	C*0704	C*0802	B*1401	B*1518	*0401	*0402	*0302	*0302
**602_A02**	UK	Het	C4A	5	3	2	A*1101	A*3101	C*0602	C*0702	B*3701	B*0702	*13	*1601	*0604	*0502
**602_B08**	UK	Het	C4A	4	2	2	A*0101	A*6801	C*0702	C*0704	B*4402	B*0702	*13	*0401	*0604	*0301
**602_B10**	UK	Het	C4A	4	2	2	A*02	A*0101	C*0304	C*07			*13	*0404	*0604	*03
**602_B11**	UK	Het	C4A	4	2	2	A*02	A*3201	C*0304	C*0102	B*4001	B*2705	*13	*1103	*0604	*0301
**602_G08**	UK	Het	C4A	4	3	1	A*0101	A*1101	C*0602	C*0602	B*3701	B*5701	*15	*0701	*0602	*0303
**602_H04**	UK	Het	C4A	4	2	2	A*2402	A*2902	C*0304	C*0702	B*4001	B*0702	*13	*0404	*0604	*0302
**604_B06**	UK	Het	C4A	4	3	1	A*02	A*02	C*0304	C*0501	B*4001	B*4402	*13	*0401	*0604	*02
**604_C10**	UK	Het	C4A	5	2	3			C*0304	C*0304	B*4001	B*4001	*1302	*801	*0604	*0402

**604_D05**	UK	Het	C4A	4	2	2	A*1101	A*6801	C*1502	C*0704			*1302	*0401	*0604	*03
**604_F05**	UK	Het	C4A	4	2	2	A*0101	A*6801	C*0602	C*0704	B*4402	B*5701	*1401	*0701	*0503	*0303
**801_A04**	UK	Het	C4A	4	2	2	A*02	A*02	C*0304	C*0202	B*4001	B*4002	*13	*11	*0604	*0301
**Sample**	**Cohort**	**Genotype†**	**Gene‡**	***C4****	***C4A*[a]**	***C4B*[b]**	**HLA-A_1**	**HLA-A_2**	**HLA-C_1**	**HLA-C_2**	**HLA-B_1**	**HLA-B_2**	**DRB1_1**	**DRB1_2**	**DQB1_1**	**DQB1_2**
**801_A10**	UK	Hom	C4A	3	1	2	A*02	A*3101	C*0304	C*07	B*4001	B*0801	*13	*03	*0604	*02
**804_A06**	UK	Het	C4A	4	2	2	A*02	A*02	C*0501	C*0704	B*4402	B*4402	*13	*0404	*0604	*0302
**804_D03**	UK	Het	C4A	5	3	2	A*02	A*02			B*4001	B*4402	*1201	*1601	*0502	*0301
**804_E04**	UK	Het	C4A	4	2	2	A*2402	A*3201	C*0304	C*0401			*1302	*1101	*0604	*0301
**806_B08**	UK	Het	C4A	4	2	2	A*02	A*02	C*0602	C*0704	B*4402	B*5701	*1301	*1302	*0604	*0603

Legend:

† Genotype of insertion

‡ *C4* gene harbouring the insertion

* Total *C4* gene copy number

[a] *C4A* gene copy number

[b] *C4B* gene copy number
