# Supplementary material for: Determination of the Loss of Function Complement C4 Exon 29 CT Insertion Using a Novel Paralog-Specific Assay in Healthy UK and Spanish Populations
Source: PLoS One. 2011 Aug 3;6(8):e22128. doi: 10.1371/journal.pone.0022128 (PMC3153930; doi:10.1371/journal.pone.0022128)
Supplement: Table S2 — C4A CT insertion CNV-SNP correlation. (PDF) [file pone.0022128.s003.pdf]

Supplementary Table S2

C4A CT insertion CNV-SNP correlation

SNP	Locus	UK		Spanish	
		R^2	Rank	R^2	Rank
RS2734331	*SKIV2L*	0.43	1	0.06	20
RS453098	*C6orf25*	0.21	2	0.01	275
RS707915	*MSH5*	0.20	4	-	-
RS707936	*C6orf27*	0.20	5	0.01	178
RS400547	*CLIC1*	0.20	3	-	-
RS9267532	*LY6G5B/CSNK2B*	0.19	6	0.008	428
RS2142234	*LY6G5B/CSNK2B*	0.19	7	-	-
RS4148876	*TAP2*	0.17	8	0.03	53
*HLA_DRB1*13*	-	0.12	11	-	-
RS3916765	Intergenic (*HLA DQB1*)	0.14	9	0.015	187
RS2228088	*TNF*	-	-	0.25	1
RS6927077	*BTNL2*	-	-	0.25	2
RS6457580	Intergenic (*BTNL2*)	0.0001	1096	0.25	3
RS11961444	*LY6G5B/CSNK2B*	-	-	0.25	4
RS6940690	Intergenic (*BTNL2*)	-	-	0.25	5
RS10947368	*HLA-DOA*	-	-	0.12	7
RS6902390	Intergenic (*HLA-DPA1*)	-	-	0.12	8
RS11964779	*MSH5*	-	-	0.12	10
RS2254083	*LY6G5B/CSNK2B*	-	-	0.12	9
